# Supplementary material for: Detailed molecular and epigenetic characterization of the pig IPEC-J2 and chicken SL-29 cell lines
Source: iScience. 2023 Feb 20;26(3):106252. doi: 10.1016/j.isci.2023.106252 (PMC10018572; doi:10.1016/j.isci.2023.106252)
Supplement: Data S2. Complete homer output for identified motifs in Chicken SL-29, related to Tables 5 and 6 — Homer motif analysis results for histone modifications H3K4me1, H3K4me3, H3K27ac, enhancers, and ATAC-seq of chicken SL-29 cell line. Parameters for possible false positives is as mentioned earlier for S5. [file mmc3.zip › Data_S2/S6/Chicken_SL_29/peak_files_enhancers/homerResults/motif13.info.html]

Motif 13

## Information for 11-CAGHNMYTCCCAC (Motif 13)

T
G
A
C
G
C
T
A
A
C
T
G
G
T
A
C
T
A
C
G
T
G
C
A
A
G
T
C
A
C
G
T
A
T
G
C
G
A
T
C
G
T
A
C
G
C
T
A
A
T
G
C
  
Reverse Opposite:
